# Association of family pharmacist continuity with patient-reported satisfaction in patients with metastatic cancer: a nationwide cross-sectional study

**DOI:** 10.1186/s40780-026-00548-4

**Published:** 2026-02-03

**Authors:** Atsunobu Sagara, Tomofumi Watanabe, Tomoya Abe, Takatsune Shimizu, Shunsuke Shirozu, Hiroyuki Terakado

**Affiliations:** 1 Division of Applied Pharmaceutical Education and Research, School of Pharmacy and Pharmaceutical Sciences, Hoshi University, 2-4-41 Ebara, Shinagawa-ku, Tokyo, Japan; 2 Department of Pharmacy, Japanese Red Cross Medical Center, 4-1-22 Hiroo, Shibuya-ku, Tokyo, Japan; 3 Department of Pharmacy, Saitama Cancer Center, 780 Komuro, Ina- machi, Kitaadachi-gun, Saitama, Japan; 4 Department of Pathophysiology, School of Pharmacy and Pharmaceutical Sciences, Hoshi University, 2-4-41 Ebara, Shinagawa-ku, Tokyo, Japan

**Keywords:** Family pharmacist, Continuous involvement, Patients with metastatic cancer, Patient satisfaction, Treatment hope

## Abstract

**Background:**

Patients with metastatic cancer face prognostic uncertainty and substantial psychological stress, requiring support for both physical and mental health. We examined the association of family pharmacist continuity with patient-reported satisfaction, and secondarily whether higher satisfaction is associated with greater treatment hope, in a nationwide sample in Japan.

**Methods:**

A nationwide online survey was conducted on 481 participants in Japan who were in contact with a pharmacist and diagnosed with metastatic cancer. The participants were categorized into groups based on the continuity of contact with their family pharmacist before and after their primary cancer diagnosis. We assessed the association of family pharmacist continuity with patient-reported satisfaction using the MISS-21J, and examined the satisfaction–treatment hope association with Spearman’s rank correlation.

**Results:**

The group with the same family pharmacist before and after the primary cancer diagnosis (Same) and the group in which the family pharmacist changed but remained involved before and after cancer diagnosis (Changed) showed significantly higher distress relief and rapport (a sense of mutual trust and understanding with the pharmacist) scores on the MISS-21J than the group without a family pharmacist from pre to post cancer diagnosis (None) (*p* < 0.001). Additionally, a strong correlation (rs = 0.740, *p* < 0.001) was observed between the MISS-21J total score and treatment hope score.

**Conclusions:**

Continuous pharmacist involvement with patients, regardless of the change in pharmacists before the primary cancer diagnosis, shows the potential to contribute to psychological support and improved treatment hope in patients with metastatic cancer. While causal inference cannot be made from this cross-sectional study, establishing a long-term support system by a family pharmacist may play an important role in promoting patient-centered cancer care.

## Background

Cancer is one of the leading causes of death worldwide [1]. Patients with metastatic cancer, who are reported to experience significant uncertainty regarding their prognosis and psychological distress [2], often experience significant mental stress and a reduction in quality of life due to limited treatment options, anxiety about physical changes, and increased pain [3, 4]. Therefore, comprehensive support from not only physicians and nurses but also pharmacists and other professionals is essential [5, 6].

The kakaritsuke-yakuzaishi (the family pharmacist) system, introduced in Japan in 2016 and often referred to as “family pharmacist” in English, provides continuous care by assigning a single pharmacist to each patient. This system aims to offer comprehensive and continuous support for medication therapy by consolidating prescriptions from multiple healthcare providers for the same patient [7, 8]. In recent years, several observational studies have begun to evaluate the effectiveness of the family pharmacist system [9–12]. In fact, despite policy efforts to promote this system, only 7.4% of the Japanese population has a family pharmacist [13]. The presence of a family pharmacist has been reported to contribute to medication support and an improved understanding of pharmacotherapy [14]; however, the effect of involvement of a family pharmacist before a primary cancer diagnosis or change in the family pharmacist after a primary cancer diagnosis on patient satisfaction and treatment hope among patients with metastatic cancer, in whom prognosis is uncertain and the psychological burden is high, have not been examined.

The Medical Interview Satisfaction Scale (MISS-29) was originally developed in the United States to assess patient satisfaction with individual doctor–patient consultations, and a shorter 21-item version (MISS-21) was subsequently adapted for use in general practice settings [15]. Based on these instruments, Japanese version of the Medical Interview Satisfaction Scale (MISS-21J) is a useful tool for evaluating patient satisfaction with pharmacists, and its reliability and validity have been confirmed in Japan [16]. MISS-21J evaluates four metrics: distress relief, communication comfort, rapport (a sense of mutual trust and understanding with the pharmacist), and compliance intent. However, the research on the use of MISS-21J for evaluating patients with metastatic cancer is scarce; therefore, the role of family pharmacist involvement in reducing psychological distress or enhancing treatment hope among such patients remains underexplored.

Therefore, this study aims to examine the association of family pharmacist continuity before and after primary cancer diagnosis with patient-reported satisfaction and, secondarily, whether higher satisfaction is associated with greater treatment hope among patients with metastatic cancer. Specifically, we focused on the first visit to the pharmacy of these patients and evaluated the continuous involvement of family pharmacists using the MISS-21J score. In addition, we examined the association between the MISS-21J score and treatment hope.

## Methods

### Survey period and participants

From January 23 to January 29, 2025, the registered monitors of a survey company (Macromill, Inc.) collected responses from 481 individuals nationwide, aged ≥ 40 years, who had contacted a pharmacist and had been diagnosed with metastatic cancer. The survey company’s monitors were randomly selected through an open recruitment process, with approximately 36 million monitors available as of January 2025. To prevent fraudulent responses, the company requires a trap survey every 6 months and an annual update of the monitor registration information [17].

### Survey items

**Fig. 1:**
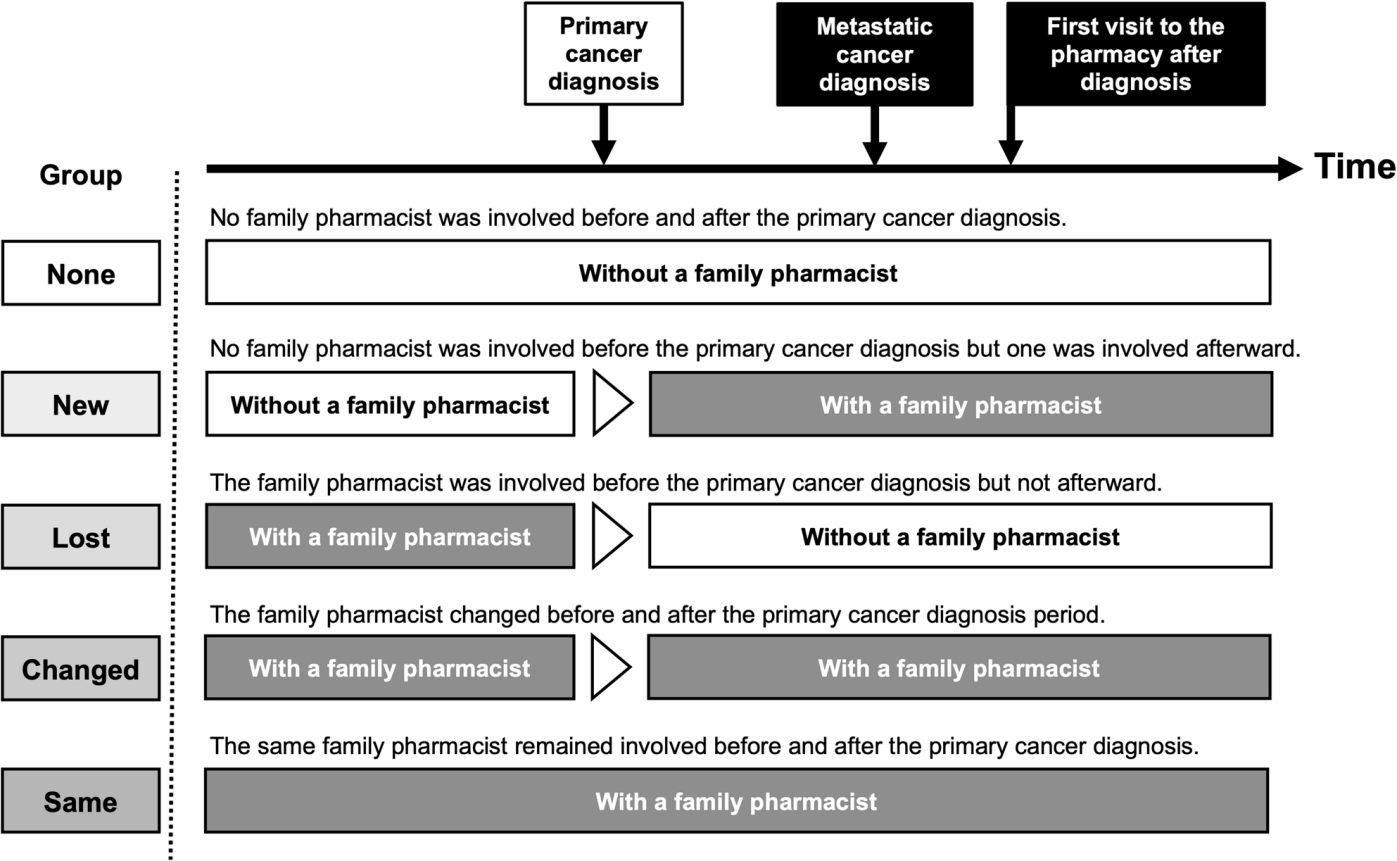
The five study groups based on whether the patients had a family pharmacist before or after their primary cancer diagnosis

The survey items are listed in Table 1. Participants’ demographic data (i.e., age, sex, and residential area) were obtained from the survey responses recorded by Macromill, Inc. In the present survey, we used the term “kakaritsuke-yakuzaishi (family pharmacist)” as it is employed in this national system. At the beginning of the questionnaire, we briefly explained that such a family pharmacist system exists in Japan, in which patients may choose one pharmacist to receive continuous care. After this explanation, participants were asked about their use of a “family pharmacist” before and after their primary cancer diagnosis. The participants were classified into five groups based on whether or not they had a family pharmacist in Fig. 1: (1) None: no family pharmacist was involved before and after the primary cancer diagnosis. (2) New: no family pharmacist was involved before the primary cancer diagnosis but one was involved afterward. (3) Lost: a family pharmacist was involved before the primary cancer diagnosis but not afterward. (4) Changed: the family pharmacist changed before and after the primary cancer diagnosis period. (5) Same: the same family pharmacist remained involved before and after the primary cancer diagnosis. The MISS-21J items were implemented based on the survey items and evaluation criteria established by Hanya et al. [16]. The questions regarding treatment hope were surveyed on a 7-point scale. In this study, “treatment hope” refers to patients’ perceived sense of encouragement and optimism regarding ongoing cancer treatment—that is, their positive motivation to continue anticancer treatments (including but not limited to chemotherapy) after a diagnosis of metastasis.

**Table 1:** Survey items

Question content		Item list	Figures
Question A	Please rate your experience with the explanations and support you received from the pharmacist at the pharmacy on a scale of 1 to 7 when you first visited the pharmacy after being diagnosed with cancer metastasis.	The questions are based on the MISS-21J study [16].	Figure 2
Question B	After the metastasis diagnosis, did your involvement with a pharmacist give you hope for treatment? Please select one option.	7. Very strongly agree	Figure 3
		6. Strongly agree	
		5. Agree	
		4. Uncertain	
		3. Disagree	
		2. Strongly disagree	
		1. Very strongly disagree	
Question C	Please select one option that applies to your use of a “family pharmacist” before and after a cancer diagnosis.	No family pharmacist was involved before and after the primary cancer diagnosis.	Figure 1
		No family pharmacist was involved before the primary cancer diagnosis but one was involved afterward.	
		The family pharmacist was involved before the primary cancer diagnosis but not afterward.	
		The family pharmacist changed before and after the primary cancer diagnosis period.	
		The same family pharmacist remained involved before and after the primary cancer diagnosis.	

### Statistical analysis

All the survey data obtained from Macromill, Inc. were compiled and analyzed. Sex and residential area were analyzed using Fisher’s exact probability test; in addition, age was analyzed using the Kruskal–Wallis test. Moreover, based on the previous reports, MISS-21J was used to calculate the average score ± standard deviation for distress relief (6 items), rapport (8 items), communication comfort (4 items), and compliance intent (3 items) as well as the overall score. MISS-21J subscale and overall scores were compared across the five continuity groups using the Kruskal–Wallis test, followed by the Steel–Dwass test for nonparametric multiple comparisons. The association between the overall MISS-21J score and treatment hope was assessed using Spearman’s rank correlation. Statistical analyses were performed using JMP Pro 17 software (SAS Institute, CA). Because this study included a relatively large sample and multiple group comparisons, we prespecified a more stringent significance level of *p* < 0.001 to reduce the risk of type I error and to focus on differences that are more likely to be clinically meaningful. This threshold did not correspond to a formal multiplicity correction but was adopted as a conservative analytic decision. Accordingly, statistical significance should be interpreted alongside effect sizes and the overall pattern of results, rather than relying on p-values alone. In interpreting the results, we therefore considered the magnitude of between-group differences and correlation coefficients, in addition to p-values.

## Results

### Patient characteristics

A total of 481 participants were analyzed (None = 204, New = 49, Lost = 25, Changed = 65, Same = 138; Table 2). Overall, 294 (61.1%) were male and 187 (38.9%) were female; sex distribution did not differ significantly across groups (*p* = 0.06). The mean age was 61.2 ± 0.48 years (mean ± SE) overall and was similar between groups (None = 62.4 ± 0.73; New = 60.1 ± 1.50; Lost = 62.0 ± 2.09; Changed = 62.0 ± 1.30; Same = 59.3 ± 0.89; *p* = 0.13). Participants resided across all major regions of Japan with no significant between-group differences in regional distribution (*p* = 0.16). These findings indicate that baseline demographics were broadly comparable among the five continuity groups.

**Table 2:** Patient characteristics

	None (*N* = 204)		New (*N* = 49)	Lost (*N* = 25)	Changed (*N* = 65)	Same (*N* = 138)	Total (*N* = 481)	*p* value
Sex								
Male/Female	121/83		22/27	16/9	46/19	89/49	294/187	0.06
Age								
Means ± SE	62.4 ± 0.73		60.1 ± 1.50	62.0 ± 2.09	62.0 ± 1.30	59.3 ± 0.89	61.2 ± 0.48	0.13
Residential Area								
Hokkaido	8		2	3	3	6	22	0.16
Tohoku	6		1	1	3	11	22	
Kanto	80		14	9	23	39	165	
Chubu	40		13	5	11	22	91	
Kinki	38		4	5	14	31	92	
Chugoku	12		7	1	0	9	29	
Shikoku	4		3	0	3	3	13	
Kyushu	16		5	1	8	17	47	

### Patient satisfaction and treatment hope

**Fig. 2:**
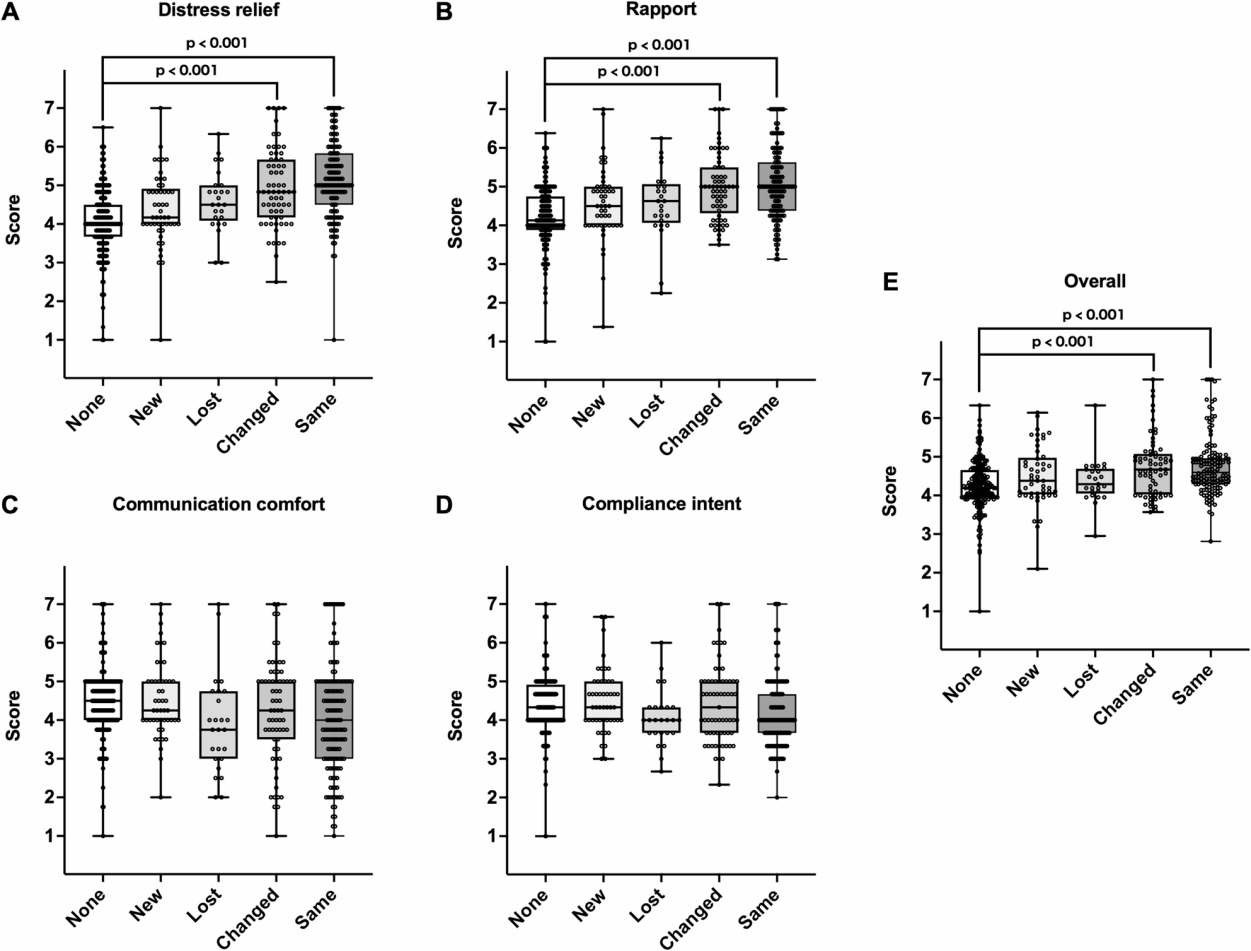
Comparison of patient satisfaction across five family pharmacist continuity groups (None, New, Changed, Same, and Lost as defined in Fig. 1), using the MISS-21J subscales. **A**) Distress relief, **B**) Rapport (a sense of mutual trust and understanding with the pharmacist), **C**) Communication comfort, **D**) Compliance intent, and **E**) Overall score of the MISS-21J. The vertical axis represents the mean scores for each MISS-21J subscale (range 1–7, with higher scores indicating greater satisfaction)

**Fig. 3:**
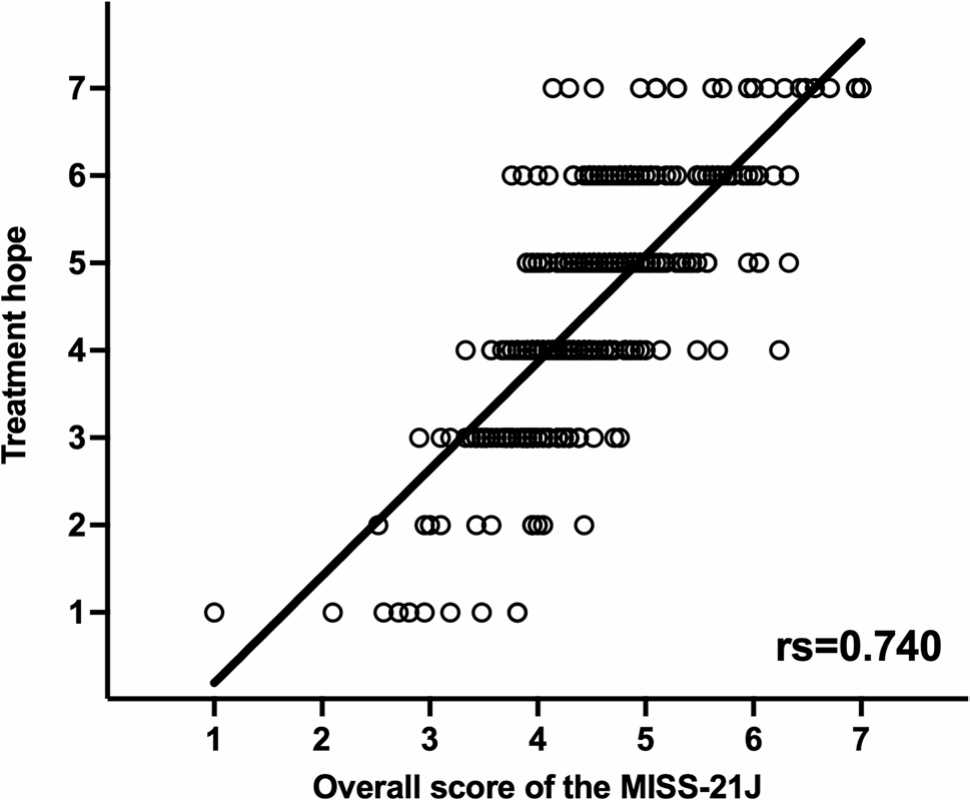
Correlation between the overall score of the MISS-21J and treatment hope. The x-axis represents the overall score of MISS-21J, and the y-axis represents treatment hope (7-point Likert scale). rs: Spearman’s rank correlation coefficient

The Same and Changed groups exhibited significantly higher MISS-21J scores for distress relief and rapport than the None group (Fig. 2A and B; *p* < 0.001). In contrast, no significant differences were observed in communication comfort and compliance intent (Fig. 2C and D). Furthermore, the Same and Changed groups showed significantly higher overall scores than the None group (Fig. 2E; *p* < 0.001). Finally, a strong correlation was observed between the MISS-21J scores and treatment hope when pharmacists were involved (Fig. 3; rs = 0.740, *p* < 0.001).

## Discussion

The results of this study suggest that the continuous involvement of family pharmacists with patients with metastatic cancer may be associated with greater distress relief and rapport, as well as higher levels of treatment hope. These results are consistent with the policy of the Japanese Ministry of Health, Labour and Welfare [8], which recommends that every Japanese citizen have a family pharmacist, and indicate that family pharmacists may be an important source of support, particularly in cases such as metastatic cancer [18], where patients suffer from significant physical and psychological burdens. Although no significant differences were observed among the five groups in terms of communication comfort and compliance intent, the Same and Changed groups showed significantly higher scores than the None group for distress relief and rapport. This suggests that the involvement of a family pharmacist may help reduce psychological burden and foster rapport among patients.

Although absolute differences in distress relief and rapport scores between continuity groups were approximately one point on the 7-point MISS-21J scale, we interpret this difference to be clinically meaningful. On this scale, a one-point change generally represents a shift of one response category (e.g., from “agree” to “strongly agree”) in patients’ perceptions of being understood, reassured, and supported by pharmacists. To our knowledge, a formally established minimally important difference for the MISS-21J has not yet been reported, such a shift is likely perceptible to patients and indicative of an improved experience with psychological support.

It is noteworthy that the Changed group exhibited significant effects similar to those observed in the Same group. This suggests that the patient’s medication information [19], treatment plan [20], and psychological background were appropriately transferred to the new pharmacist. In addition, patients in the Changed group may have carried forward their previous positive experience of receiving support from a pharmacist. Thus, the quality of inter-pharmacy collaboration and the psychological support provided by the “continuity of the pharmacist’s presence” [21] may have contributed to the reconstruction of rapport. In contrast, no significant effect was observed in the New group, in which patients were assigned a family pharmacist for the first time after their primary cancer diagnosis; this suggests that building trust and embracing psychological support require some time. These results suggest that pharmacists may play a potential role not only as a “person in charge” but also a “constant source of support” for patients [22], and that this potential role may be particularly important at major turning points during treatment, such as the metastatic diagnosis. Furthermore, the strong positive correlation (rs = 0.740) between the overall MISS-21J score and treatment hope suggests that higher patient satisfaction may be associated with greater treatment hope. However, because both variables were assessed at the same time point using self-report Likert scales, this association also appears related to shared method variance. We cannot exclude the possibility that individuals with higher inherent treatment hope tend to report greater satisfaction in their interactions with pharmacists. It is presumed that pharmacists’ comprehensive understanding of patients’ health conditions [23] and treatment progress, as well as their continuous support, may be associated with patients feeling secure and maintaining a positive attitude toward treatment; however, this potential mechanism cannot be confirmed in this cross-sectional study.

In this study, our primary focus was on the association between family pharmacist continuity and patient-reported satisfaction, and, secondarily, on the association between satisfaction and treatment hope. Conceptually, we assumed that any influence of family pharmacist continuity on treatment hope would operate mainly through improvements in patients’ experiences of psychological support and satisfaction with pharmacist consultations. For this reason, we did not formally test a direct association between continuity and treatment hope in the present analysis, and we consider that such mediation pathways would be better evaluated in future longitudinal studies.

This study is the first to examine the relationship between family pharmacist involvement and psychological outcomes in patients with metastatic cancer. Most previous studies on family pharmacist intervention have focused on patients with chronic diseases, such as hypertension or diabetes; thus, detailed analyses of metastatic cancer patients are limited [24]. Therefore, the findings of the present study are useful for demonstrating the significance of family pharmacists’ involvement in treating patients with metastatic cancer. However, this study has several limitations. Design: its cross-sectional nature precludes causal inference, and we cannot rule out reverse causation (for example, that patients with greater inherent treatment hope or perceived support are more likely to continue seeing a family pharmacist and to report higher satisfaction). In addition, our assessment of family pharmacist continuity was based on participants’ self-reported use of a “kakaritsuke-yakuzaishi (family pharmacist)” and did not verify formal registration status under the national system. Although we used the term as it is defined and commonly employed in national policy and public communication, patients’ awareness and understanding of the system may vary. Some respondents may have interpreted “family pharmacist” more broadly as a pharmacist at their usual pharmacy with whom they have an ongoing relationship, regardless of official designation. Such variation in interpretation could have led to exposure misclassification of the continuity variable, and this possibility should be kept in mind when interpreting the magnitude of the observed associations. Measurement: uncertainty remains due to unmeasured confounding, including performance status and depressive symptoms, which were not fully captured. Data: although the sample size was relatively large for a study of patients with metastatic cancer, it may still have been insufficient for stable subgroup analyses by region or age. In addition, participants were recruited from a large nationwide online survey panel, which may limit the generalizability of our findings. Individuals who enroll in such panels are likely to have higher digital literacy and may differ from the broader metastatic cancer population in Japan in terms of socioeconomic status, treatment setting, and access to healthcare services. Furthermore, metastatic cancer diagnosis was self-reported and not verified against clinical records, so some misreporting or misunderstanding of the term “metastatic cancer” cannot be excluded. These factors could have influenced the representativeness of our sample and should be kept in mind when interpreting the results. Finally, the survey format could not fully characterize the quality of the pharmacist–patient relationship or the specifics of the interventions provided (e.g., frequency, intensity). Nevertheless, the study’s strength lies in providing nationwide insight into the psychological-support dynamics among patients with metastatic cancer, and the findings may inform future policy design and clinical practice.

## Conclusions

Having a family pharmacist may be associated with relieving distress and fostering rapport among patients with metastatic cancer and may be related to higher levels of treatment hope. To extend these benefits, it is important to raise public awareness and encourage more Japanese citizens to engage with a family pharmacist. Establishing a system in which pharmacists play a central role in coordinating care and supporting patients as a part of the healthcare delivery system promoted by Japan’s Ministry of Health, Labour and Welfare is expected to contribute significantly to improving the quality of cancer care and realizing a patient-centered support system.

## Data Availability

The datasets used and/or analysed during the current study are available from the corresponding author on reasonable request.

## Abbreviations

MISS-21J: Japanese version of the Medical Interview Satisfaction Scale
